# Seasonal Variations of Mercury Levels in Selected Medicinal Plants Originating from Poland

**DOI:** 10.1007/s12011-016-0645-z

**Published:** 2016-02-29

**Authors:** M. Ordak, M. Wesolowski, I. Radecka, E. Muszynska, M. Bujalska-Zazdrozny

**Affiliations:** 1 Department of Analytical Chemistry, Medical University of Gdansk, Gdansk, Poland; 2 Department of Pharmacodynamics, Centre for Preclinical Research and Technology (CePT), Medical University of Warsaw, Warsaw, Poland; 3 Chair and Department of Psychiatry, Medical University of Warsaw, Warsaw, Poland; 4 Department of General Biology, Medical University of Bialystok, Bialystok, Poland

**Keywords:** Mercury, Medicinal herbs, Seasonal variations of mercury levels

## Abstract

The presence of mercury in the living cells may be caused by environmental pollution with this element, which is referred to as a toxic xenobiotic. Many literature reports have provided evidence for toxic effects of low levels of mercury in the human body. Therefore, it seems essential to investigate mercury content in food and in natural environment, particularly its seasonal variations. The objective of this study was to determine trace amounts of mercury in 45 samples of 20 medicinal plant species collected in northern Poland, in various seasons of the year, i.e., in autumn 2012 and then spring 2013. The results obtained showed that the levels of mercury in the herbs were lower in spring (3.66–34.89 ng/g) than in autumn (4.55–81.54 ng/g). The statistically significant correlation (*p* < 0.05) between the levels of mercury in herbs collected in spring and autumn indicates hazardous accumulation of the element in plants in autumn. The highest levels of mercury were found in leaves and plants growing in the vicinity of busy streets. Perennials plants have a significantly higher mercury levels as compared to those of monocarpic plants. Furthermore, commonly used herbal plants have a significantly higher mercury levels as compared to those less common.

## Introduction

Research into the chemical composition and pharmacological action of raw medicinal plant materials has confirmed the advantageous use of herbs in the prevention and treatment of numerous ailments. Apart from active substances, i.e., macro- and microelements that are indispensable for health, medicinal plants may introduce toxic elements to the human body and impair its normal function. Although raw plant materials undergo quality control, their contamination with heavy metals has been reported [1, 2]. Therefore, it seems justified to monitor the elemental composition of raw medicinal plant materials used in phytotherapy [3].

Mercury is one of the elements which in the living cells are considered to be dangerous xenobiotics. Usually, plants absorb mercury from soil through the root system and to a lesser extent through leaves, directly from the air [4–9]. In soil, mercury occurs in various chemical forms and is entrapped mainly in macromolecular humus substances. This element is available for plants in the amounts proportional to those released from soil, only in the methylmercury form. Mercury is easily transported through roots to the aerial parts of plants, where it is deposited in necrotic tissues, such as periderm and wood [10, 11]. It has been shown that some plants are capable to translocate microelements from leaves to twigs and branches, just before the leaf fall period. Sparing the indispensable elements, plants also accumulate toxic mercury. The level of mercury in the plant should not be higher than 20 ng/g [10, 12].

According to some authors, mercury obtained from the so-called dry deposition can be rinsed out from the surface of living plants, e.g., by atmospheric precipitation [13, 14]. However, throughout the vegetation period, the level of mercury in plant tissues increases both due to dry and wet deposition, more intensely than in the surrounding soil. This is also caused by mercury mobility in the air-soil-plant system and its accumulation by certain plant species. It has been demonstrated that appropriate agrotechnical procedures can largely decrease mercury absorption by plants [5, 6, 15, 16].

Up to now, research into seasonal variations in mercury concentration and deposition has shown a marked relationship of its environmental content with its levels in the atmospheric air and surrounding soil. Studies conducted in an area of Atlantic Forest at Ilha Grande Island located in the south-east of Brazil showed substantial fluctuations of mercury in the vegetation season, depending on its content in the duff formed by tree leaves exposed to high mercury pollution. Industrial works situated close to the Island are responsible for the emission of mercury to the atmosphere. The mercury content in the leaves of trees that dominate in that region is the highest between June and August (225 ± 17 ng/g), and the lowest between December and May (94 ± 54 ng/g), depending on deposition of this element originating from duff (fallen leaves) and precipitation intensity. Similar results have been reported in studies on the magnitude of mercury deposition in the atmosphere over China and on the content of other elements in tree leaves in the south of Sweden. The soils are Hg contaminated in two ways: atmospheric deposition and leaching through the soil profiles of Hg-organic matter complexes [10, 15, 17, 18]. Atmospheric Hg may account for all of the Hg deposited in litterfall [12]. It has been found that mercury accumulation in plants increases with time. Chronic deposition leads to mercury arrest mainly in roots [5, 19]. Skinner et al. showed that four species of aquatic plants exposed to mercury for 30 days accumulated and bound the mercury within the roots [20]. The content of mercury in plants is the highest during the day, being twofold lower at night [21]. Other authors have found a significant correlation between vegetation period and the concentration of mercury in the leaves of several medicinal plants. The concentration of mercury in the tested leaves increased in the period of enhanced photosynthesis [22].

Mercury accumulation in medicinal plants is a particularly unfavorable phenomenon [23, 24]. Since not many literature data are available on the content of mercury in medicinal plants, the objective of the current study was to determine and assess its seasonal variations in medicinal herbs. There are no reports in the literature which examined whether there is significant influence of vegetation and utility on concentration of mercury in medicinal plants which were collected at different seasons of the year.

## Materials and Methods

### Plant Material

A set of 45 samples obtained from 20 medicinal plant species was analyzed. The plants had been collected in northern Poland in autumn 2012 and spring 2013. Taxonomic classification of the plants was verified based on a dictionary of environmental science. Samples were collected in the area of Tri-city (Gdansk, Sopot, Gdynia), mainly in forests, as well as in allotment and kitchen gardens. The plants were collected at the same sites in autumn and spring. The content of mercury in the soil depends on interplay of many factors. The most important role is played by air temperature, amount of rainfall, and such soil characteristics as its pH, aeration, content of organic compounds, and certain elements, e.g., nitrogen, iron, calcium, and phosphorus in the soil. Medicinal plants species used in the study were derived from areas characterized by different soil conditions. These were allotments and kitchen gardens located at different distance from the street and forest as well. Detailed data on the samples and collection sites are presented in Table 1.

**Table 1:** Analyzed plant material

Sample number	Plant species	Collected morphological part	Place of harvest	Therapeutic effect
1	*Petroselinum sativum* (*Parsley*)^a, c^	Leaves	Allotments, 30 m from the street	*Diureticum*, *carminativum*, *stomachicum*, *spasmolyticum*
2				
3	*Hedera helix L.* (*Ivy*)^b, d^	Leaves	Home garden, 20 m from the street	*Expectorans*, *sedativum*, *rubefaciens*, *antirheumaticum*, *antipyreticum*
4				
5	*Urtica dioica L.* (*Nettle*)^a, c^	The whole plant	Forest	*Diureticum*, *antirheumaticum*, *haemostaticum*, *alterans*, *rubefaciens*
6				
7	*Convolvulus arvensis L.* (*Field bindweed*)^a, d^	Roots	Forest	*Purgans*, *depurativum*, *antisepticum*, *hypotonicum*, *spasmolyticum*
8		Leaves		
9	*Sambucus nigra L.* (*Elderberry*)^b, c^	Fruits	Forest	*Expectorans*, *diaphoretic*, *diureticum*
10		Leaves and flower buds		
11	*Anethum graveolens L.* (*Dill*)^a, c^	Leaves and flowers	Allotments, 30 m from the street	*Stomachicum*, *carminativum*
12				
13	*Rosa canina L.* (*Wild rose*)^b, c^	Leaves	Home garden, 20 m from the street	*Alterans*, *cholagogum*, *diureticum*, *adstringens*, *tonicum*
14		Fruits		
15		Leaves		
16		Flowers		
17	*Lavandula officinalis Chaix* (*Common lavender*)^b, c^	Flowering tops of shoots	Home garden, 20 m from the street	*Diureticum*, *cholagogum carminativum*
18				
19	*Bellis perennis* (*Daisy*)^b, d^	The whole plant	Allotments, 30 m from the street	*Depurativum*, *alterans*, *diureticum*
20				
21	*Solidago gigantea Aiton* (*Giant goldenrod*)^b, d^	Flowering tops of shoots	Forest	*Epurativum*, *antirheumaticum*, *hypotonicum*
22				
23	*Taxus baccata L.* (*Yew*)^b, d^	Leaves	Park	*Antihelminticum*
24		Leaves		
25		Fruits		
26	*Altheae rosea L. Cav.* (*Alcea rosea*)^a, d^	Flowers	Home garden, 30 m from the street	*Emolliens*, *protectivum*
27				
28	*Rubus idaeus L.* (*Raspberry*)^b, c^	Leaves	Allotments, 30 m from the street	*Diureticum*, *adstringens*
29		Fruits		
30		Leaves		
31	*Rubus fruticosus L.* (*Blackberry*)^b, c^	Leaves	Allotments, 30 m from the street	*Diureticum*, *expectorans*
32		Fruits		
33		Leaves		
34	*Mentha piperita L.* (*Pepper mint*)^b, c^	Leaves	Allotments, 30 m from the street	*Cholagogum*, *digestivum*, *antisepticum*
35				
36	*Salvia officinalis L.* (*Sage*)^b, c^	Leaves	Home garden, 10 m from the street	*Antidiabeticum*, *alterans*, *antiphlogisticum*
37				
38	*Capsella bursa-pastoris* (*Shepherd*’*s purse*)^a, d^	The whole plant	Home garden, 20 m from the street	*Haemostaticum*, *spasmolyticum*, *antipyreticum*
39				
40	*Lamium album L.* (*White dead-nettle*)^b, d^	Flowering tops of shoots	Forest	*Expectorans*, *haemostaticum*, *emolliens*
41				
42	*Monarda didyma* (*Bee balm*)^a, d^	Leaves	Home garden, 10 m from the street	*Antisepticum*, *stomachicum*
43				
44	*Viscum album L.* (*Mistletoe*)^b, d^	Leaves and young branches	Home garden, 10 m from the street	*Hypotonicum*, *vasotonicum*, *diureticum*, *haemostaticum*
45				

^a^Polycarpic plant

^b^Monocarpic plant

^c^Commonly used plant

^d^Less common plant

The harvested medicinal plants were dried at room temperature, in an airy place to reduce a high water content that could have initiated adverse biological processes. After drying, foreign material such as other plant parts, weeds, waste, soil particles, and dust were manually separated. The dried up plants were placed in paper bags that were stored till analysis in a dry, dark place, with no exposure to sun rays.

### Determination of Mercury

A Varian SpectrAA 250 Plus absorption/emission spectrometer (Varian, Australia) was used to quantify mercury concentration using cold vapor technique (Cold Vapor Atomic Absorption Spectrometry, CVAAS). In this technique, mercury contained in a sample is reduced to its elemental form. Next, using argon stream, mercury is washed out from the solution in the form of vapors and moved to an absorption cuvette, where it absorbs the radiation emitted by a cathode mercury lamp [24–26]. The calibration graph was determined based on the analysis of standard solutions, containing: 10, 20, 50, 75, and 90 ng Hg in 1 mL of solution. The graph was linear and described by equation *A* = 0.011 × c and *R*^2^ = 0.9959.

A wet-digestion of the samples was carried out according to a procedure developed previously [27], with slight modifications. The weighed amount (1 ± 0.05 g) of the plant material was transferred to a Teflon vessel. Next, 5 mL of redistilled water obtained from a quartz apparatus Heraeus Quarzglas (Destamat®, Germany) and 5 mL of concentrated (65 %) HNO_3_ (Selectipur®, Darmstadt, Merck) were added. The vessel was tightly closed, put aside for 3 min, and then vortexed. Wet digestion was performed in a high-pressure microwave mineralizer UniClever MB-1z (Plazmatronika, Wrocław, Poland) in three steps, using the parameters shown in Table 2. The cooling time of a sample after wet digestion, before opening of the cap, was 10 min. The solution was placed in a volumetric flask and diluted with redistilled water up to 25 mL.

**Table 2:** Terms of mineralization of analyzed samples

Mineralization step	Time (min)	Power microwave generator (%)	Minimum pressure (atm.)	Maximum pressure (atm.)	Temperature (°C) [min-max]
I	6	50	17	20	150–180
II	5	85	32	35	150–180
III	5	100	38	40	180–200

The accuracy of the method was assessed using a certified reference material, white cabbage leaves BCR® – 679 (IRMM, Geel, Belgium), with a declared mercury content of 6.3 μg/kg. The analyte recovery was 100.32 ± 0.95 %. To check the precision of mercury measurements, absorption of two randomly chosen solutions was determined, for which 12 repetitions were performed. The relative standard deviations, RSD, were 0.74 and 1.7 %, respectively. Thus, it was stated that the measurements were accurate and precise.

### Statistical Analysis

Each sample was analyzed in triplicate and the results were presented as the arithmetic mean (M) with standard deviation (SD). All concentrations of mercury in the plant samples were expressed in ng/g of dry mass (d.m.). The statistical analysis was performed using the IBM SPSS Statistics 22 software, at a *p* < 0.05 significance level. The Student’s *t* test for independent samples was applied to check for statistically significant differences in the mercury content in the plant materials, depending on the season of the year. The Student’s *t* test for single sample was employed to compare the results with the norms established by the Ministry of Health [28]. Correlations between the levels of mercury in the particular parts of plants were determined by correlation analysis.

## Results

### Mercury in Medicinal Plants

The analysis of mercury content in medicinal plants showed its range to be 3.66–81.54 ng/g. The highest content of mercury (81.54 ng/g) was found in autumn in the leaves of yew (sample 23), whereas the lowest in spring in white dead-nettle blossoming tops (3.66 ng/g, sample 41). High concentration of mercury was observed in daisy (sample 19, 68.54 ng/g). The content of mercury in nettle (sample 6) did not exceed 5 ng/g. A similar low content of mercury was found in leaves and blossom of *Anethum graveolens* (sample 12).

**Fig. 1:**
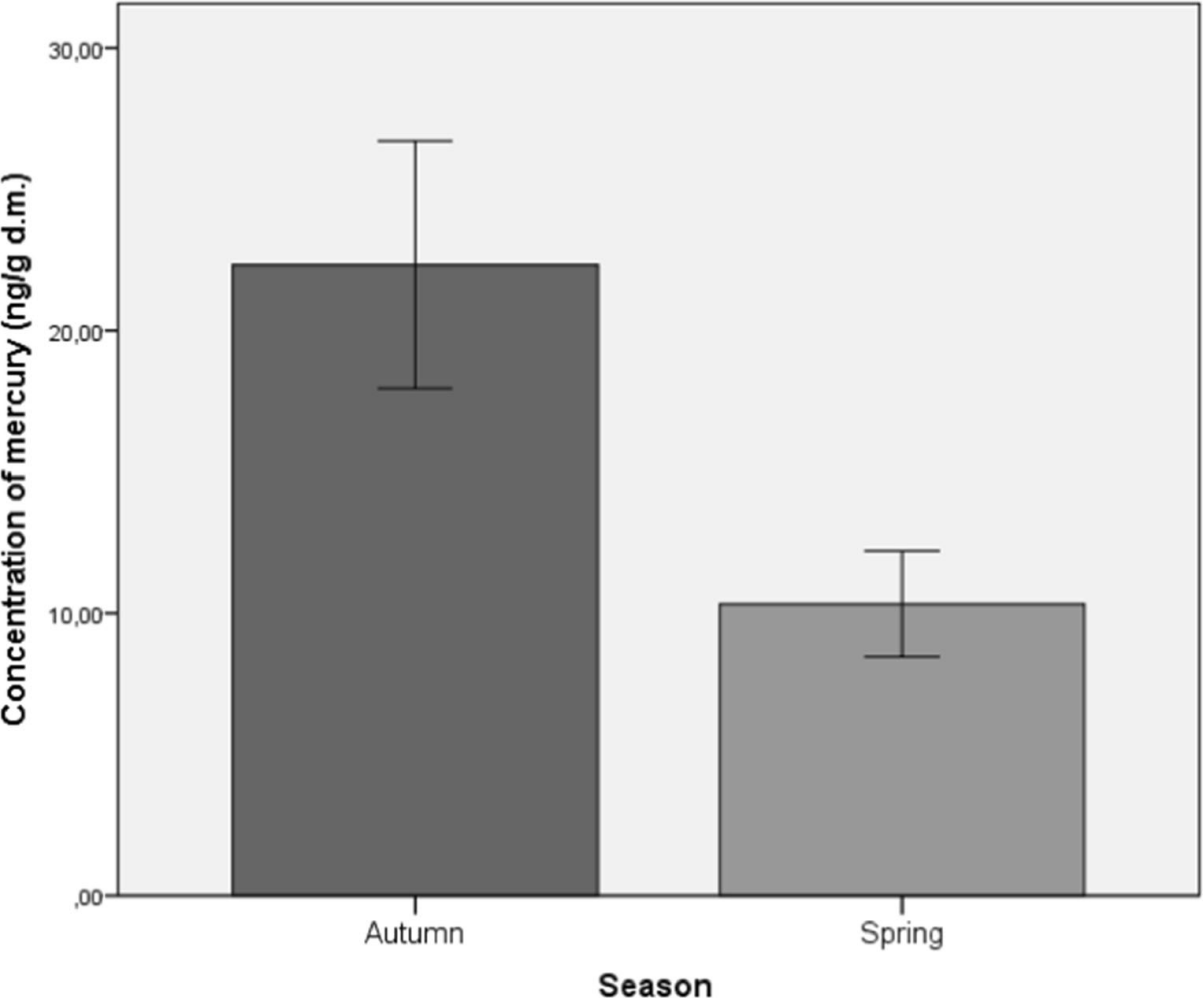
Average concentration of mercury in the analyzed medicinal herbs (*M* ± SD)

Statistically significant differences were found in the content of mercury between the plant materials collected in spring and autumn (*t* (31.11) = 2.66; *p* < 0.05). Figure 1 presents the mean content of mercury in medicinal plant with dispersion of the results. The mean level of mercury in the medicinal herbs and dispersion of the results were higher in autumn (*M* = 22.33; SD = 4.13) than in spring (*M* = 10.36; SD = 1.78). The difference was either slight, as in the case of blossoming sprout tops of giant goldenrod (samples 21, 22), or large, e.g., in the leaves of yew (samples 23, 25). In autumn months, more extreme results or deviations were obtained.

The comparison of mercury levels in various morphological parts of the plants showed that the content of this element exhibited the greatest fluctuations in leaves and herbs. A slight dispersion of the results around the mean was observed in fruits and inflorescences. Furthermore, the leaves of ivy (samples 3, 4), bee balm (samples 42, 43), yew (samples 23, 25), raspberry (samples 28, 30), blackberry (samples 31, 33), wild rose (samples 13, 15), and sage (samples 36, 37) were found to be the highest contaminated with mercury. The content of this toxic element in leaves ranged from 5.84 ng/g (sample 33) to 81.54 ng/g (sample 23). High content of mercury was also found in daisy whose all parts are used as a medicinal herb and in yew fruits (sample 24; 27.63 ng/g). High levels of this element were also detected in the flowers of *Alcea rosea*—27.90 ng/g (sample 26), whereas in the roots of field bindweed (sample 7), the content of mercury was low (6.67 ng/g).

**Fig. 2:**
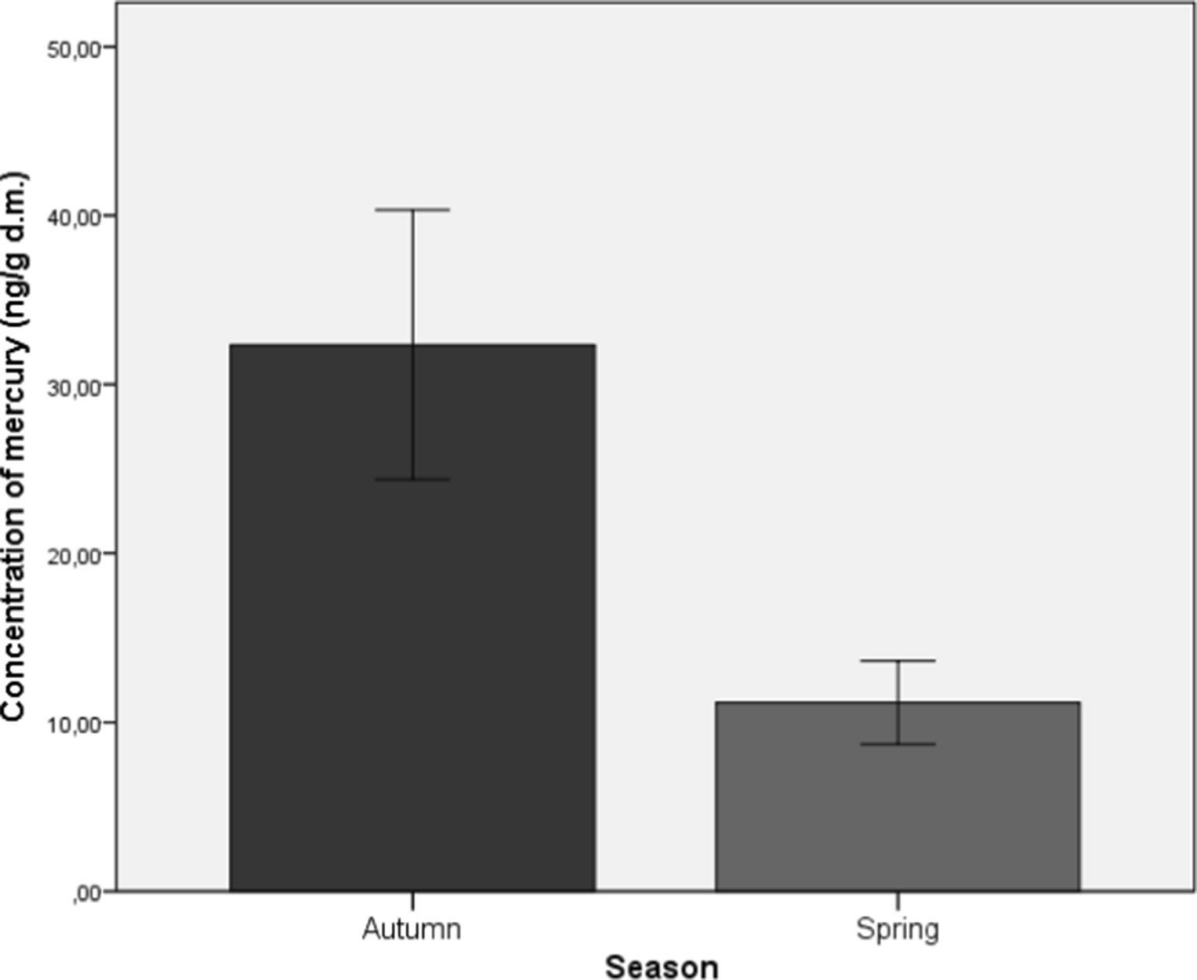
Average concentration of mercury in leaves of the analyzed medicinal herbs (*M* ± SD)

The Student’s *t* test showed statistically significant differences in the levels of mercury only in leaves (Fig. 2) collected in spring and autumn (*t* (9.52) = 2.67; *p* < 0.05). In the leaves collected in autumn, the mercury level was significantly higher (*M* = 32.35; SD = 7.59) as compared to that in the spring months (*M* = 11.17; SD = 2.35).

The levels of mercury in the herbs studied were compared to the norms established by the Ministry of Health [28]. According to the regulation, mercury content should not exceed 10 ng/g in food products for children and 30 ng/g in dried medicinal and culinary herbs. The level of mercury in plants collected in spring appeared to be significantly lower than the accepted norm (*t* (17) = 4.38; *p* < 0.001). However, the elevated content of mercury in autumn was not high enough to achieve a statistically significant difference (*t* (17) = 1.29; *p* > 0.05).

### Collection Site of Plants

The results of this study showed a massive effect of the collection site (home gardens, allotments, forest) on the mercury content. Medicinal herbs collected in the autumn in allotments and home gardens exhibited a significantly higher level of mercury (*M* = 24.63, SD = 4.54) compared to the plants collected in this season of the year in the forest (*M* = 11.37; SD = 3.78), (t (7.3) = 2.48; *p* < 0.05). The highest levels of mercury were found in herbs collected in home gardens and allotments located in the close vicinity of places exposed to substantial pollution (busy streets, construction sites). These herbs included daisy (samples 19, 20), leaves of ivy (samples 3, 4), and leaves of yew (samples 23, 25). However, the level of mercury was lower in samples harvested in natural environment, i.e., in forests or allotment gardens, where air pollution is probably the lowest. This can be easily exemplified by roots and leaves of field bindweed (samples 7, 8) and the leaves of parsley (samples 1, 2).

### Correlation Analysis

**Fig. 3:**
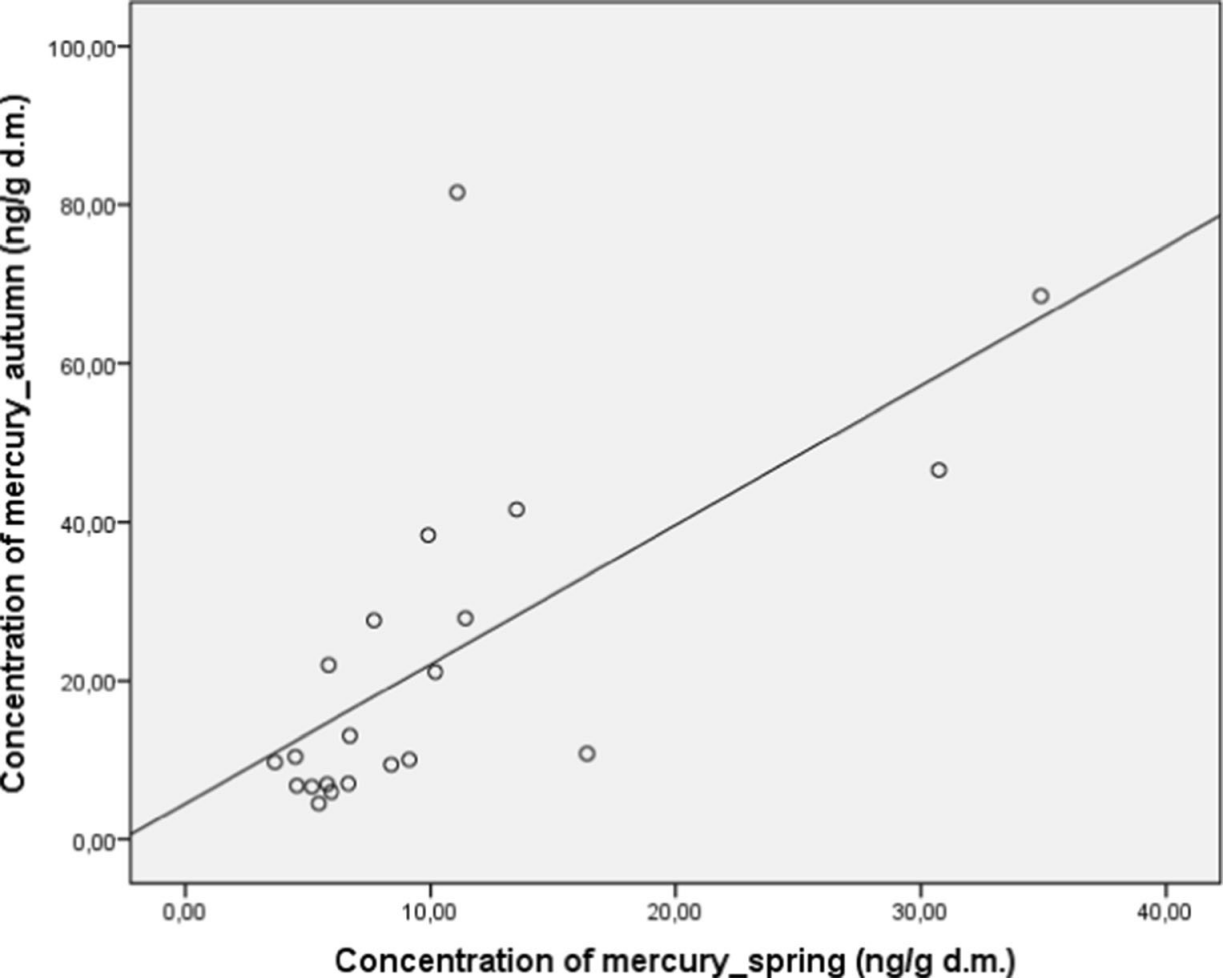
Correlation between concentration of mercury in autumn and spring in medicinal herbs

The correlation analysis was employed to determine the relations between the levels of mercury in the medicinal herbs studied. The analysis revealed a significant positive and strong correlation between the levels of mercury in herbs collected in spring and autumn (*r* = 0.66; *p* < 0.05; Fig. 3).

**Fig. 4:**
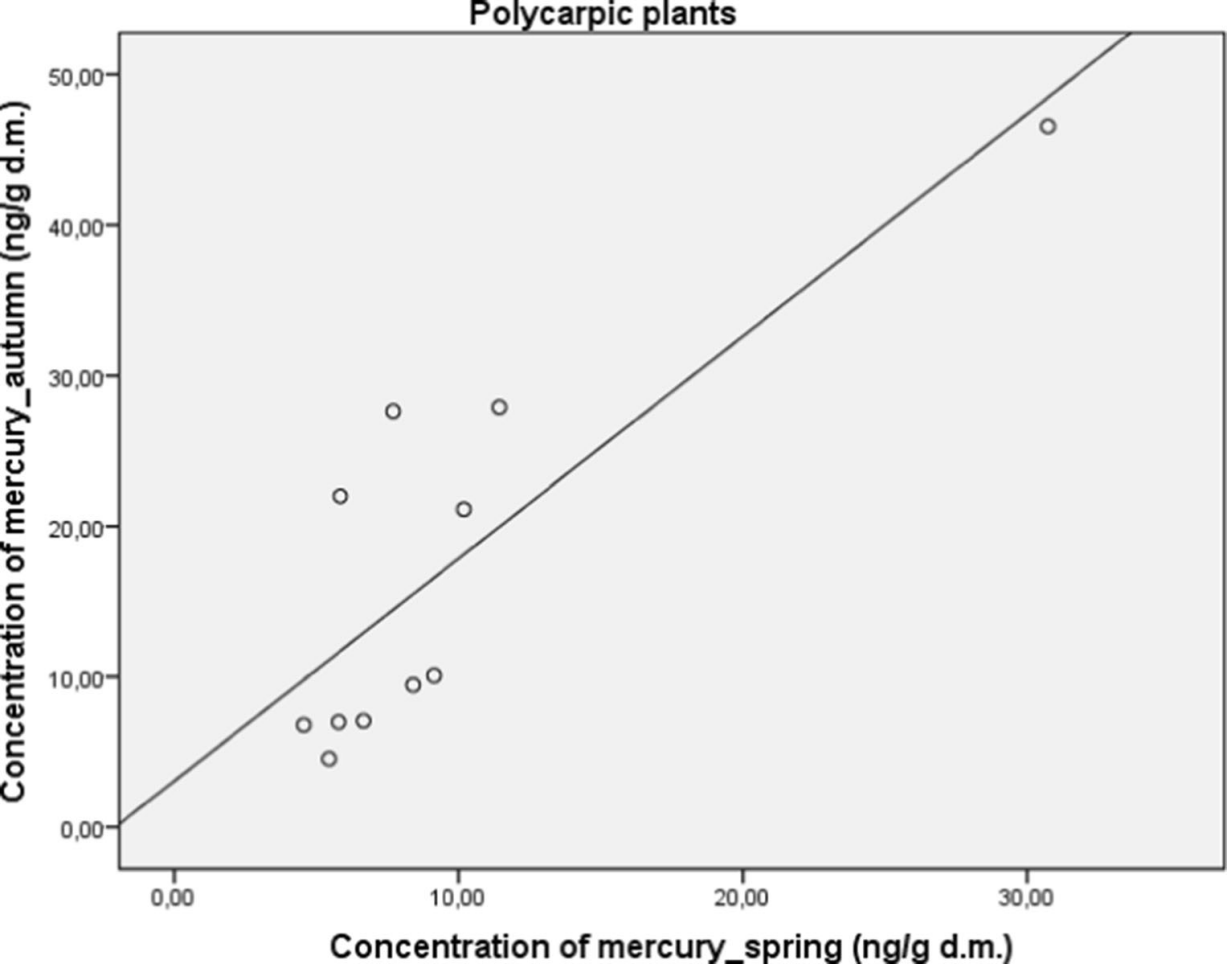
Correlation between concentration of mercury in autumn and spring in polycarpic plants

**Fig. 5:**
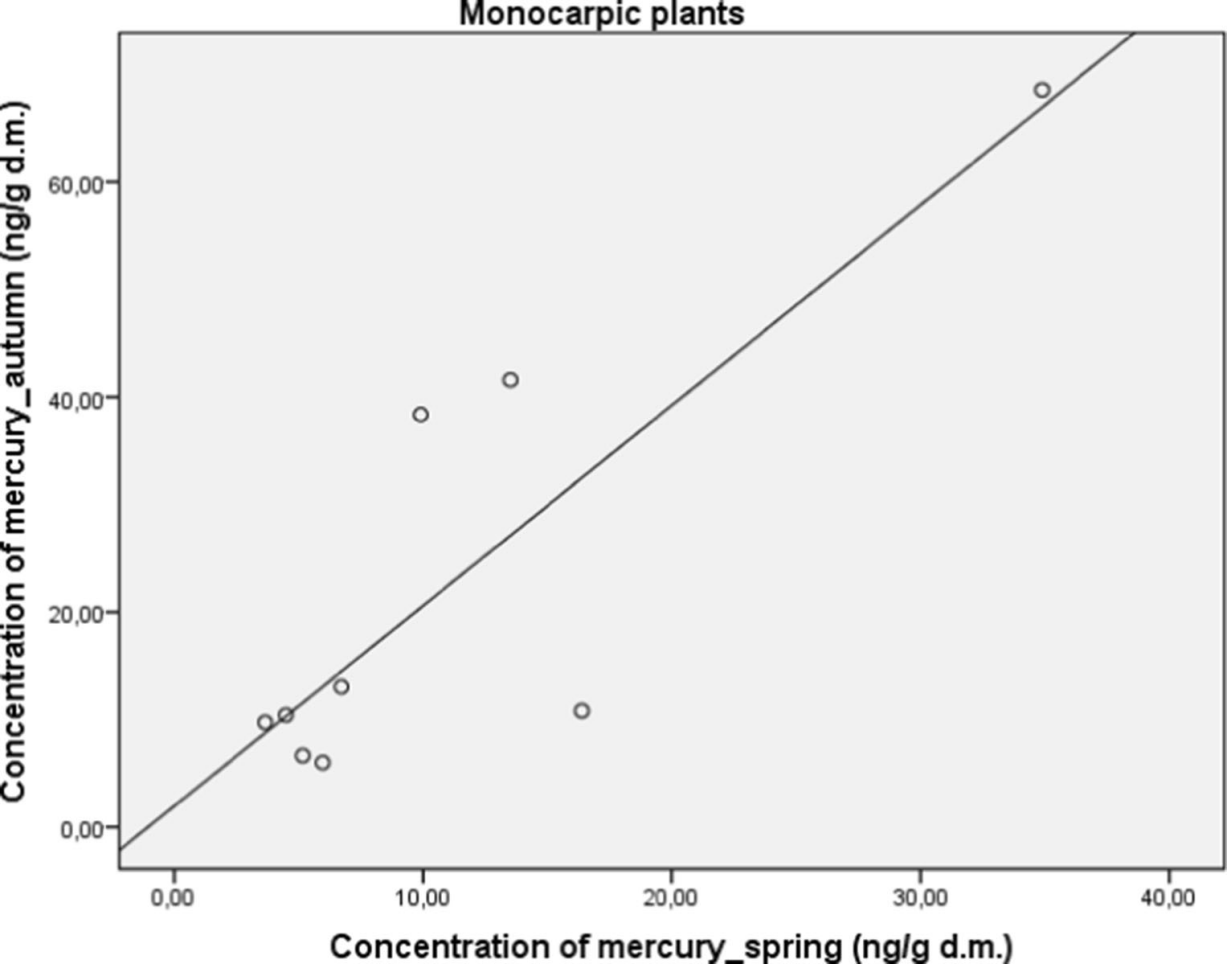
Correlation between concentration of mercury in autumn and spring in monocarpic plants

Herbal plants were also divided into another subgroups, namely polycarpic (perennials) and monocarpic (biennials or annuals). Perennials plants have a significantly higher concentration of mercury (*M* = 22.99; SD = 7.35) in comparison to biennials and annuals (*M* = 5.22; SD = 0.71), (*t* (7.13) = 2.41; *p* < 0.05). A significant and strong correlation between concentration of mercury in autumn and spring in both subgroups was found for polycarpic (*r* = 0.82; *p* < 0.05; Fig. 4) and monocarpic (*r* = 0.84; *p* < 0.01; Fig. 5) plants.

**Fig. 6:**
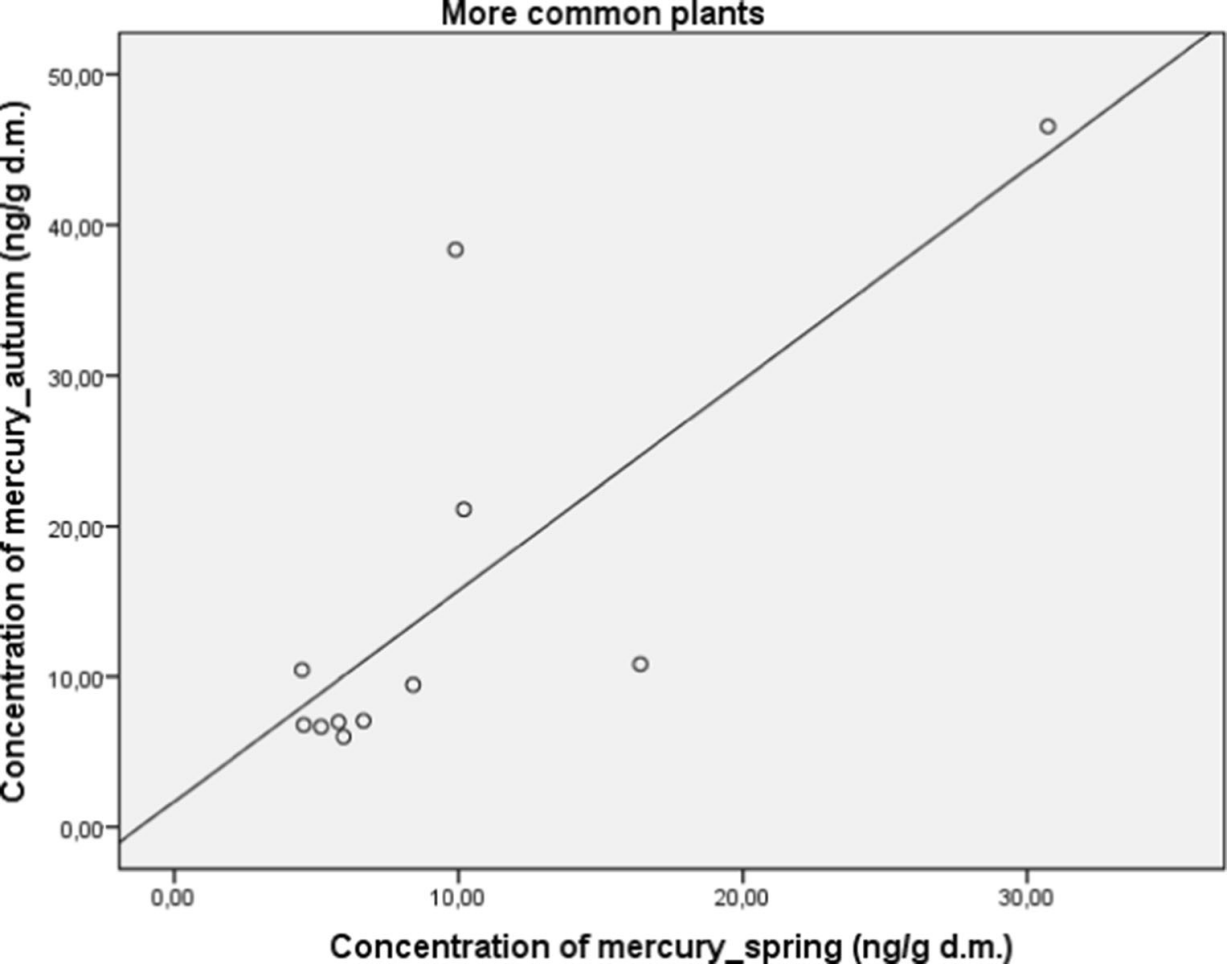
Correlation between concentration of mercury in autumn and spring in more common plants

**Fig. 7:**
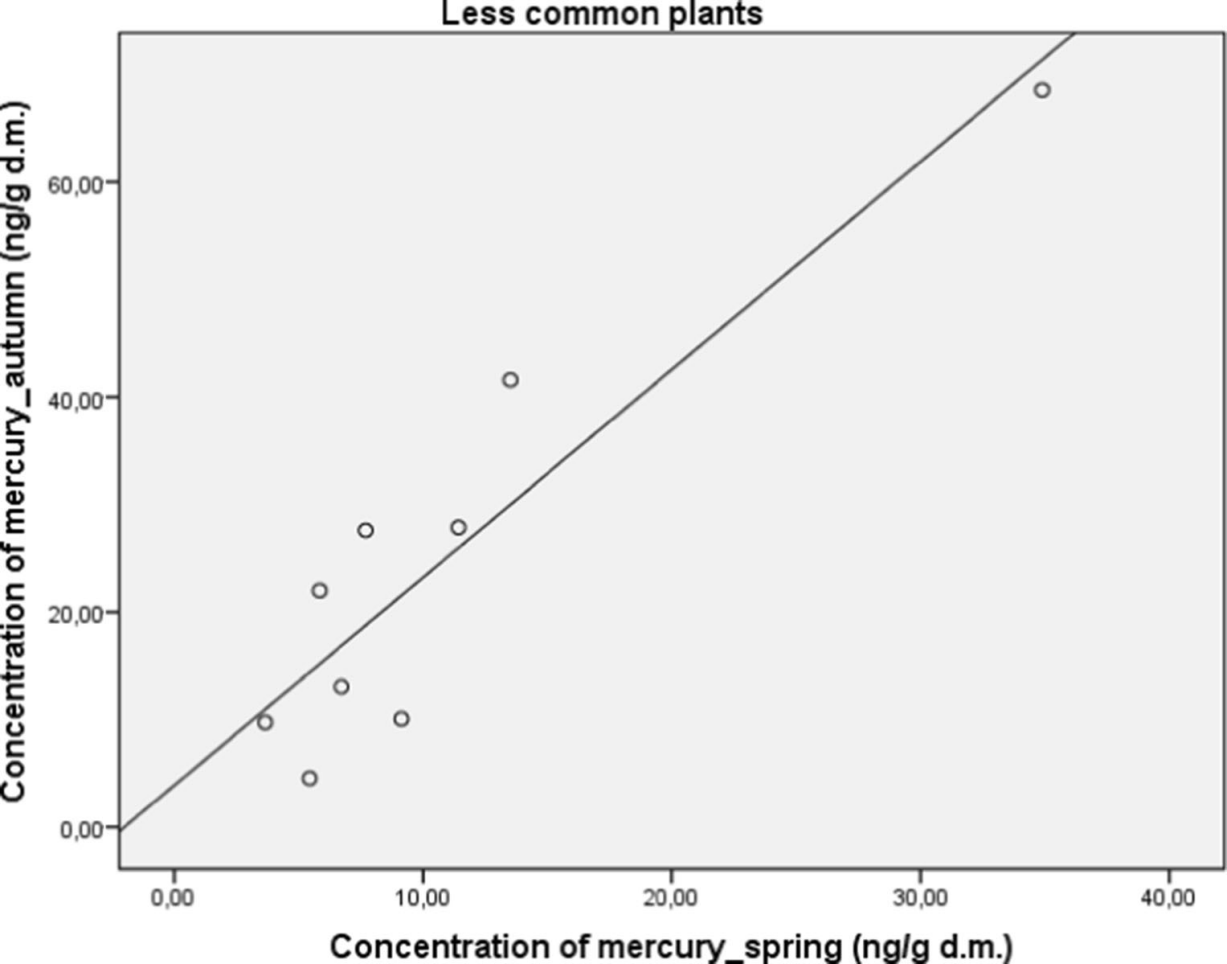
Correlation between concentration of mercury in autumn and spring in less common plants

Herbal plants were also divided into the more or less common ones. Those which are more common have anti-inflammatory and diuretic activities among others. More commonly used herbal plants have a significantly decreased concentration of mercury (*M* = 10.23; SD = 1.23) in comparison to those less common (*M* = 19.95; SD = 4.6), (*t* (22.84) = 2.04; *p* < 0.05). A significant and strong correlation between concentration of mercury in autumn and spring in both subgroups was found for more common (*r* = 0.77; *p* < 0.05; Fig. 6) and less common (*r* = 0.92; *p* < 0.01; Fig. 7) plants.

Moreover, an attempt has been made to learn whether there is a significant relationship between the temperature at which the plants were collected and the concentration of mercury. The average temperature at which particular herbs were collected was established at 13.8 ± 1.72 °C in the spring and 21.37 ± 1.79 °C in the autumn. A significant correlation has been between the concentration of mercury in whole plants, leaves, inflorescences, and the temperature at which they were collected for leaves (*r* = 0.61; *p* < 0.01), inflorescences (*r* = 0.87; *p* < 0.05), and whole plants (*r* = 0.42; *p* < 0.01).

## Discussion

Except for cadmium and lead, mercury is included to heavy metals unnecessary for living organisms. Beyond a certain limit, it is toxic and hazardous to plants, animals, and humans. Sparse literature data reveal that mercury can accumulate in human tissues due to the use of plant materials for therapeutic purposes. Herbal preparations with an increased content of toxic elements may become an additional source of their intake by human organism, and along with their beneficial effect, they can intoxicate the organism. The results of this study seem to indicate that the content of this toxic element in herbs depends on the following:

- Plant species

Among 20 species of medicinal plants analyzed in this study for the mercury presence, it was found that the greatest predisposition for accumulation of this element was demonstrated by yew and the lowest by white dead-nettle. This probably results from the fact that the yew, as a perennial plant, deposits mercury accumulated by the roots in the leaves in subsequent vegetation seasons. The fact that the yew does not shed the needles for winter causes an increase in the mercury level in these organs [29]. In turn, white dead-nettle is an annual plant, and therefore, the time for mercury accumulation by this plant is shorter than the long time of the yew. Similarly, other annual plants such as dill and stinging nettle demonstrated small amounts of this element in the examined organs. Some plants characterized by high levels of mercury in their tissues produced deformed roots (daisy) and the leaves with chlorotic and brown spots (yew). Similar toxic effects of mercury on plants were described by other authors [18, 19].

b) Degree of soil and atmosphere contamination

In this study, the plants derived from relatively unpolluted sites, cultivated in soils where applicable, appropriate agricultural practices are applied, for example, in allotments, contained lower amounts of mercury compared to the species growing wild, harvested near busy streets. The amount of mercury uptaken from the soil by the root system of the plant depends on the degree of this element accumulation and capacity of its exclusion from the circulation through the soil sorption complex [30]. Soil properties such as high pH, high content of floatable fraction (mostly colloidal clay), and humus are the factors inhibiting mercury accumulation by the plants. Then, mercury is bound by an appropriate buffer system of the soil and becomes not assimilable for plant roots.

Furthermore, ionic relations of such elements as nitrogen, iron, calcium, and phosphorus in soil determine either the reduction (antagonism) or promotion (synergism) of absorption of mercury by plants [31]. Plant roots absorb mercury along with other substances from the soil. If there are no such competing substances, mercury absorption is relatively greater. The forest is an example of the natural ecosystem, in which the ecological balance is preserved. Probably favorable soil conditions in forest inhibited the accumulation of mercury in these plants.

A significantly higher level of mercury in medicinal herbs collected in the autumn in allotments and home gardens located in the close vicinity of busy streets compared to the plants collected in the forest could be due to unfavorable conditions of soils where the plants were grown or due to localization of allotments and kitchen gardens near streets; therefore, these plants were exposed to exhaust gases. The above-mentioned factors promote the accumulation of mercury in plants. A similar phenomenon has been observed in Poland when evaluating the content of Hg in soils of the Silesian-Cracow region [32]. Most of this metal was found in the surface layers of city parks’ soil.

c) Season of the year of plants harvesting

Statistical analysis of the results confirmed a significant relationship, both in the group of polycarpic and monocarpic plants, between the metal concentration and season of the year. The higher was mercury concentration in both groups of plants in the spring, the higher was it in autumn. This research seems to indicate that the plants uptake mercury during the whole vegetation season, starting in spring, summer, and ending in autumn. Therefore, higher concentration of that element in the organs of examined species is found in autumn. Concurrently observed a statistically significant correlation between mercury concentration in herbs harvested in spring and autumn proves the dangerous phenomenon of mercury accumulation in plants in the autumn period.

It is worth mentioning that during the collection of plans for the study, in the spring and in the autumn, there was a relatively scant rainfall. Less rainfall was observed in the spring in comparison to the autumn. Along with rainfall, as a result of wet deposition, one can observe a drop of concentration of mercury on the soil surface and on the appropriate plant strata in the form of Hg^2+^ ion [12]. It is evidenced by the results presented in this paper, stating that in the autumn, there is a significantly higher level of mercury in the plants as compared to that in the spring.

The results of this study also showed that the higher the temperature at which the plants were collected, the higher the concentration of mercury in leaves, inflorescences, and whole plants. This is probably related to evaporation of elemental mercury from soils [33]. This process is more intense on warm days. Then, the reduction of mercury in the presence of organic matter and photolytic reactions with organic and other soil components reaches the highest intensity. During this process, the elemental Hg released to the atmosphere may be absorbed by both soil surface and plants.

d) Kind of plant organ used in phytotherapy

The analysis of mercury content in particular organs of 20 species of medicinal plants showed that the highest amounts of this element are accumulated in the leaves. This is probably related to the fact that the source of heavy metals for plants may be situated beyond the soil atmosphere. The amount of metal accumulated on the leaves mainly depends on the surface of the leaf blade which deposits dust from emission, weather conditions, amount of precipitation, as well as the direction and strength of the wind. This is confirmed by the literature data, which indicate diversified ability of plants to accumulate mercury, even within the same species. This was confirmed by the analysis of mercury content in common dandelion, where the mean concentration of the element was 5 ng/g in stem, 14 ng/g in leaves, 10 ng/g in blossom, and 12 ng/g in roots [34]. Moreover, the analysis of mercury content in corn showed a greater accumulation of mercury in leaves (49 ng/g) than in seeds (10 ng/g) [35].

e) Length of vegetation period of specified plant species

Significant differences in the examined plants were observed in the range of mercury concentration. Perennial herbs (polycarpic ones) exhibit significantly higher mercury levels compared to monocarpic herbs (annual or biennial). This is confirmed by the results of other authors, which show that the level of this element accumulation in plants increases over the time [5, 18]. Similar results were obtained by examining mercury deposition in the atmosphere over China, as well as by analyzing the content of that element in the leaves of trees in southern Sweden [10, 12, 15, 17, 18].

f) Utility of medicinal plants

Due to their therapeutic effects, some plant species are more frequently used by people in phytotherapy and other ones less frequently. Significantly lower mercury concentrations were found in the raw plant material of these species compared to the plants more rarely used in phytotherapy. This may result from the fact that these species are the most commonly cultivated in gardens, on the soils where various agricultural practices are used to improve soils physical and chemical properties. Such activities restrict heavy metals uptake by the plants [5, 6, 15, 16]. At the same time, it can be assumed that Hg content in the examined herbs derived from Polish pharmacies is safe and does not pose a risk to health. It should be concluded that plant raw materials which are often derived from natural sites are exposed to environmental pollutants. It is therefore necessary not only to analyze the content of active substances in the herbs that determine their therapeutic effects but also toxic and potentially toxic elements, e.g., mercury.

## Conclusions

Comparing concentration of mercury in the studied medicinal herbs depending on the season of collection to the results in the literature, it was concluded that the results did not differ. This study indicates that concentration of mercury in the medicinal herbs depends on season of the year and place where they grow. The highest concentration of this element is accumulated in autumn and near busy roads. Taking into consideration morphological parts of the plants, highest concentration of mercury was found in leaves. Significant correlation between concentration of mercury in the medicinal herbs collected in autumn and spring suggests dangerous phenomenon of accumulation of this element. A stronger tendency to accumulation of mercury is shown by polycarpic and more commonly used plants in comparison to monocarpic and less common ones.
